# Mendelian randomization analysis reveals higher whole body water mass may increase risk of bacterial infections

**DOI:** 10.1186/s12920-024-01950-3

**Published:** 2024-07-09

**Authors:** Peng Yan, Jiahuizi Yao, Ben Ke, Xiangdong Fang

**Affiliations:** Department of Nephrology, The Second Affiliated Hospital, Jiangxi Medical College, Nanchang University, Nanchang, 330000 China

**Keywords:** Mendelian randomization, Body water mass, Infections, Sepsis, Pneumonia, UTIs, Skin infections

## Abstract

**Background and purpose:**

The association of water loading with several infections remains unclear. Observational studies are hard to investigate definitively due to potential confounders. In this study, we employed Mendelian randomization (MR) analysis to assess the association between genetically predicted whole body water mass (BWM) and several infections.

**Methods:**

BWM levels were predicted among 331,315 Europeans in UK Biobank using 418 SNPs associated with BWM. For outcomes, we used genome-wide association data from the UK Biobank and FinnGen consortium, including sepsis, pneumonia, intestinal infections, urinary tract infections (UTIs) and skin and soft tissue infections (SSTIs). Inverse-variance weighted MR analyses as well as a series of sensitivity analyses were conducted.

**Results:**

Genetic prediction of BWM is associated with an increased risk of sepsis (OR 1.34; 95% CI 1.19 to 1.51; *P* = 1.57 × 10^− 6^), pneumonia (OR: 1.17; 95% CI 1.08 to 1.29; *P* = 3.53 × 10^− 4^), UTIs (OR: 1.26; 95% CI 1.16 to 1.37; *P* = 6.29 × 10^− 8^), and SSTIs (OR: 1.57; 95% CI 1.25 to 1.96; *P* = 7.35 × 10^− 5^). In the sepsis and pneumonia subgroup analyses, the relationship between BWM and infection was observed in bacterial but not in viral infections. Suggestive evidence suggests that BWM has an effect on viral intestinal infections (OR: 0.86; 95% CI 0.75 to 0.99; *P* = 0.03). There is limited evidence of an association between BWM levels and bacteria intestinal infections, and genitourinary tract infection (GUI) in pregnancy. In addition, MR analyses supported the risk of BWM for several edematous diseases. However, multivariable MR analysis shows that the associations of BWM with sepsis, pneumonia, UTIs and SSTIs remains unaffected when accounting for these traits.

**Conclusions:**

In this study, the causal relationship between BWM and infectious diseases was systematically investigated. Further prospective studies are necessary to validate these findings.

**Supplementary Information:**

The online version contains supplementary material available at 10.1186/s12920-024-01950-3.

## Introduction

Infections increase the risk of hospitalization and death. It is reported that about 20% of global deaths are attributed to infections [1]. With antibiotic resistance, new pathogens, and an aging population, the burden of infections is expected to continue to increase. Therefore, it is critical to identify modifiable risk factors for these infections.

Body water mass (BWM) may be altered in the early stages of disease development and influence body water distribution, which makes BWM an attractive predictor of preclinical disease. Thanks to simple, safe, affordable, and easily accessible bioimpedance measurements, numerous studies have investigated the relationship between body water-fluid imbalance and infectious disease outcomes in various populations. For example, in studies of patients with viral liver disease, HIV, bacteremia, and dialysis peritonitis, a strong correlation has been found between body water content and the risk of poor prognosis [2–5]. Observational studies have shown that optimizing hydration prevents bacteremia and urinary tract infections [6]. It has also been suggested that overhydration is independently affecting the survival of critically ill septic patients [7]. Inferring causality from available data is challenging due to the susceptibility of observational studies to confounding and reverse causality.

Mendelian randomization (MR) is a tool that uses genetic variation as a relevant risk factor to estimate the causal effect of certain factors on an observed outcome [8]. Recently reported anthropometric components such as body mass index (BMI) have been extensively studied and demonstrated to be associated with the risk of infections [9, 10]. However, the impact of BWM on infection risk has not been addressed. Here, we applied MR analysis to determine the potential causal effect of BWM levels on the risk of five common infections, including sepsis, pneumonia, urinary tract infections (UTIs), intestinal infections (IIs), and skin and soft tissue infections (SSTIs).

## Methods

### Genetic instrument for BWM

Instrumental variables associated with BWM levels were obtained from a genome-wide association study (GWAS) of 331,315 European-origin individuals in Neal’s laboratory (http://www.nealelab.is). We selected 418 SNPs as genetic variables at the BWM level with a genome-wide significant threshold *P* value of less than 5 × 10^− 8^ and a linkage disequilibrium (LD) threshold of *R*^*2*^ < 0.001 and kb = 10,000. The instruments statistics for BWM are shown in Supplementary Table 1. The genetic instrument was estimated to explain 3.5% of the phenotypic variance in BWM levels and had an *F*-statistic of 71.2. The relevant equations are as follows, R^2^ = β2 (1-EAF)*2EAF and F = (N-2)*R^2^/(1-R^2^) [11]. All BWM levels were obtained by bioelectrical impedance analysis using the Tanita BC418MA Body Composition Analyzer and were accurate to 0.1 kg.

### Outcome data sources

Outcome-related data were obtained from large biomedical databases. The relevant data above are summarized in supplementary Table 2. In brief, the summary-level data for sepsis (GWAS ID: ieu-b-4980, including 11,643 cases and 474,841 control subjects), pneumonia (GWAS ID: ieu-b-4976, including 22,567 cases and 463,917 control subjects), and UTIs (ieu-b-5065, including 21,958 cases and 464,256 control subjects) were available from the UK Biobank Consortium, which are available in the Integrated Epidemiology Unit (IEU) OpenGWAS program (mrcieu.ac.uk) by GWAS ID. When no available SNPs were found in an outcome GWAS, we retrieved and used proxy SNPs (R^2^ ≥ 0.8 with BWM-associated original SNPs). We also collected summary statistics from the FinnGen consortium (Release 9, https://r9.finngen.fi/) for several common infections, including intestinal infections, GTI in pregnancy, SSTIs, and sepsis and pneumonia subtypes. For outcome SNPs, we used Steiger filtering to remove SNPs associated with outcomes stronger than exposure [12].

We also explored the causal relationship between BWM and several common edematous disorders, including chronic kidney disease (CKD), type 2 diabetes (T2D), heart failure (HF) and hypothyroidism. GWAS data for CKD were obtained from an analysis of up to 480,698 Europeans by Wuttke et al [13]. We also obtained joint-sex genetic information on diabetes from Angli Xue et al [14].

### Statistical analysis

To satisfy the validity of instrumental variables (IVs) in MR analyses and the unbiased estimation of causal effects, the following three core assumptions need to be complied with: (1) the variable must be strongly correlated with the exposure of interest; (2) it must not be associated with confounding factors between each exposure and outcome; and (3) only pass-through exposures affect outcome [15].

For the two-sample MR analysis, inverse-variance weighted (IVW) random-effects models were applied to assess MR effect values. There is a potential for horizontal pleiotropy as genetic variation is used, especially when it affects the results through biological members independent of the BWM axis. We applied a variety of sensitivity analyses to assess horizontal pleiotropy, including weighted median [16], MR-Egger [17], and MR Pleiotropic Residuals Sum and Outliers (PRESSO) [18]. The weighted median method delivers enhanced causal estimates if at least half of the weights are from ineffective SNPs. MR-Egger is a multidirectional method that corrects for potential horizontal pleiotropy, while the MR-PRESSO method primarily targets and adjusts for potential outliers. Implementation of the above three models can provide complementary support to IVW models for assessing causality and pleiotropy. Cochran’s Q and MR-Egger’s intercepts were used to estimate heterogeneity and potential pleiotropy between the different instruments, respectively. Cochran Q to quantify the heterogeneity of the individual causal effects, with a *P* value ≤ 0.05 indicating the presence of pleiotropy, so a random effects IVW MR analysis should be used [19]. When MR-Egger’s intercept shows *P* > 0.05, indicating no evidence of potential horizontal pleiotropy. Multivariable MR analysis was performed to assess the impact of underlying pathologic edema factors on the association between BWM and infections [20]. Adjustment for correlated traits with shared genetic predictors and for known pleiotropic factors is possible in Multivariable MR analyses, which are statistical methods that allow associations of genetic variation with multiple risk factors to be included in the analysis. As with the previous MR analysis, the main parameters in the MVMR analysis are set by default, such as the LD threshold set *R*^*2*^ < 0.001and kb = 10,000.

Multiple tests for correlations between BWM and the thirteen outcome traits were performed using the Bonferroni correction as a heuristic. A *P* value of < 0.0038 (0.05/13) was considered strong evidence. All MR analyses were performed using the “TwoSampleMR” package (version 0.5.6), “MRPRESSO” (version 1.0), and the “MVMR” package (version 0.4) of the R software (version 4.3.0).

### Ethics statement

This study is based on previously publicized data and does not involve new studies with human or animal subjects. The datasets used in the current analysis were all available in publicly available databases and did not require individual ethical approval or informed consent. This study was submitted in accordance with the 2021 Reporting of Observational Studies Using Mendelian Randomization to Enhance Epidemiology (STROBE-MR) guidelines [21].

## Results

### MR analysis for correlation between BWM and risk of infection

**Fig. 1:**
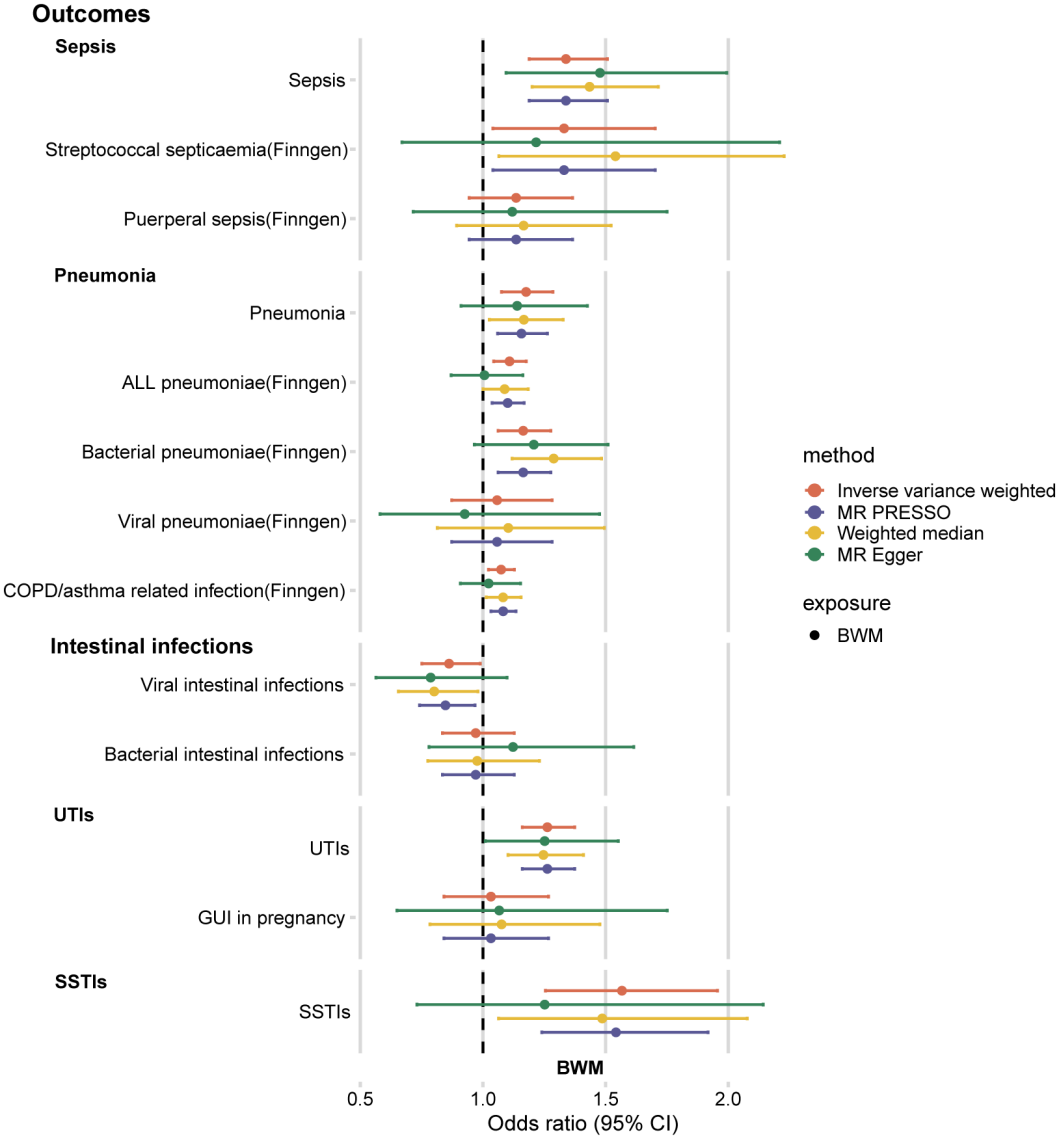
Results of MR analysis examining the effect of BWM on the risk of infections. BWM, Body Water Mass; UTIs, Urinary tract infections; GTI in pregnancy, genitourinary tract infection in pregnancy; SSTIs, skin and soft tissue infections

We performed MR analysis to examine the association between BWM levels and the risk of five infections. Genetically predicted higher BWM levels were positively associated with the risk of sepsis (odds ratio (OR) per standard deviation (SD) increase in BWM: 1.34; 95% CI 1.19 to 1.51; *P* = 1.57 × 10^− 6^), pneumonia (OR: 1.17; 95% CI 1.08 to 1.29; *P* = 3.53 × 10^− 4^), UTIs (OR: 1.26; 95% CI 1.16 to 1.37; *P* = 6.29 × 10^− 8^), and SSTIs (OR: 1.57; 95% CI 1.25 to 1.96; *P* = 7.35 × 10^− 5^). Suggestive evidence indicates that BWM is negatively associated with viral intestinal infections (OR: 0.86; 95% CI 0.75 to 0.99; *P* = 0.03) (Supplementary Table 3) (Fig. 1). These findings were directionally consistent in sensitivity analyses including MR-Egger, weighted median, and MR-PRESSO. MR-Egger analysis suggested no indication of horizontal pleiotropy (intercept *P* > 0.05) (Supplementary Table 4). The association between BWM and sepsis, pneumonia, UTIs, viral intestinal infections, and SSTIs persisted in MR-PRESSO analyses after 0, 1, 0, 1, and 3 possible outliers were detected and removed, respectively.

To minimize sample overlap, for the subgroup analysis of sepsis, we selected the streptococcal sepsis and postpartum sepsis datasets from the FinnGen consortium (Release 9) for repeated analyses. The results showed association between genetically predicted BWM and risk of streptococcal sepsis (OR: 1.33; 95% CI 1.04 to 1.70; *P* = 0.02), but not with puerperal sepsis (OR: 1.14; 95% CI 0.94 to 1.37; *P* = 0.18) (Fig. 1). Similarly, we selected the pneumonia-related GWAS from the FinnGen database as the outcome. The results showed that genetically predicted BWM was positively associated with all pneumonia (OR: 1.11; 95% CI 1.04 to 1.77; *P* = 7.66 × 10^− 4^), bacterial pneumonia (OR: 1.16; 95% CI 1.06 to 1.28; *P* = 1.25 × 10^− 3^), and COPD/asthma-associated infections (OR: 1.07; 95% CI 1.02 to 1.13; *P* = 0.004), but not with viral pneumonia (OR: 1.06; 95% CI 0.87 to 1.28; *P* = 0.57) (Supplementary Table 5). No evidence of multiplicity points to these results.

In addition, we analyzed COVID-19 data from the covid-19 Host Genetic Program. The results showed an association between genetically predicted BWM and severe covid-19 (OR: 1.22; 95% CI 1.06 to 1.40; *P* = 0.003), hospitalized covid-19 (OR: 1.24; 95% CI 1.14 to1.37; *P* = 3.23 × 10^− 6^), and critical covid-19 (OR: 1.15; 95% CI 1.11 to 1.20; *P* = 7.85 × 10^− 13^) (Supplementary Table 5). Supplementary Fig. 1 visualizes the results.

There was no strong evidence of an association between BWM levels and bacterial intestinal infections (OR: 0.97; 95% CI 0.84 to 1.13), and GUI in pregnancy (OR: 1.03; 95% CI 0.84 to 1.27) (Fig. 1).

### MR analysis for correlation between BWM and risk factors

**Fig. 2:**
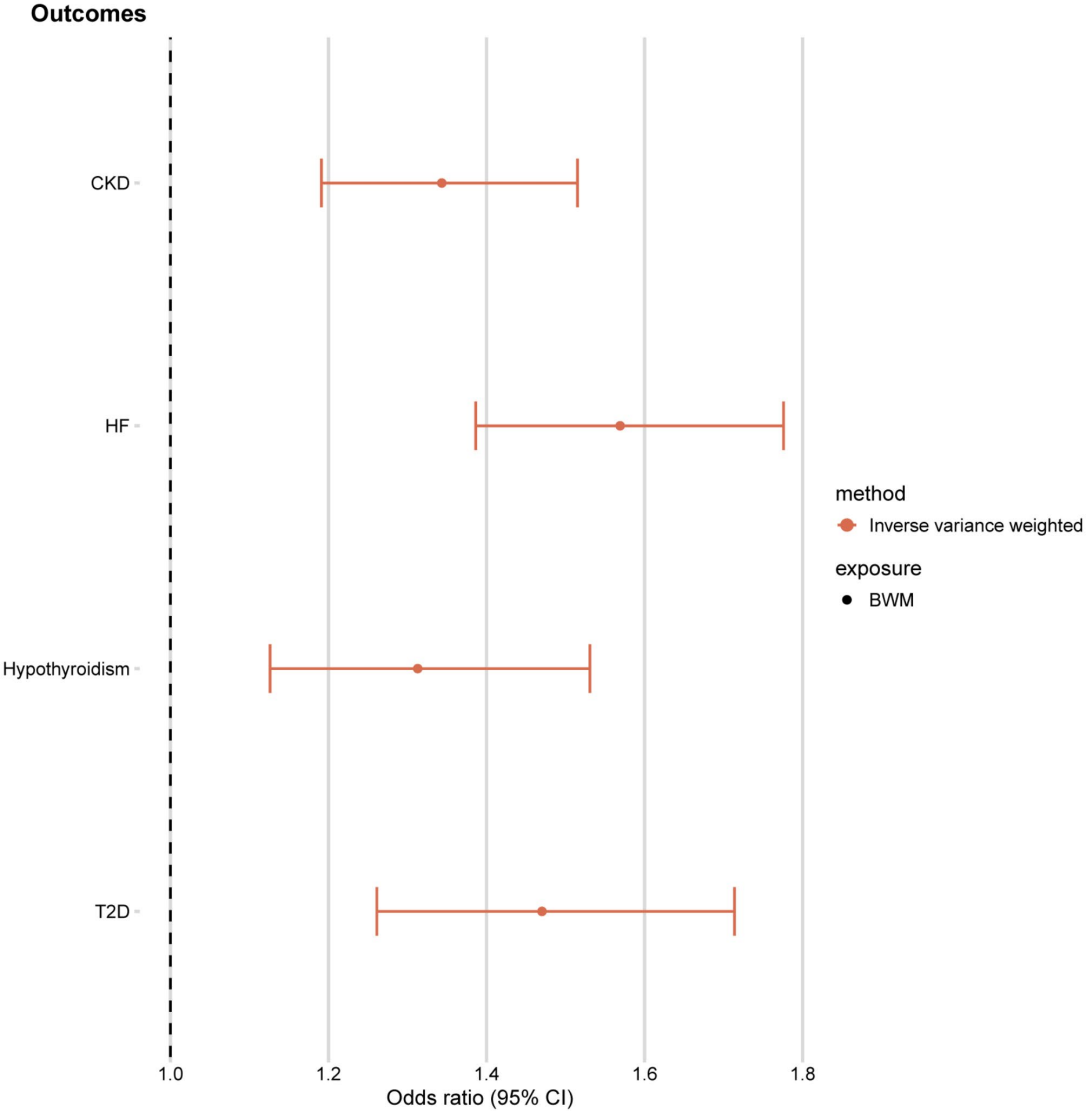
Results of MR analysis examining the effect of BWM on the risk of several common edema disorders BWM, Body Water Mass CKD, Chronic Kidney Disease T2D, Type 2 Diabetes HF, Heart Failure

Considering that whole BWM often accompanies edema disorders, we also explored the causal relationship between genetically predicted BWM and several common edema disorders. There was robust or suggestive evidence that genetically higher levels of BWM were associated with higher risk of CKD (*P* = 8.32 × 10^− 7^), heart failure (*P* = 9.98 × 10^− 13^), type 2 diabetes (*P* = 5.10 × 10^− 4^), and hypoparathyroidism (*P* = 1.51 × 10^− 6^) (Fig. 2). There was no evidence of pleiotropy in the MR-Egger analysis (MR-Egger intercept, *P* > 0.05) (Supplementary Table 6).

### Multivariable MR analysis of the relationship between BWM and infections

**Fig. 3:**
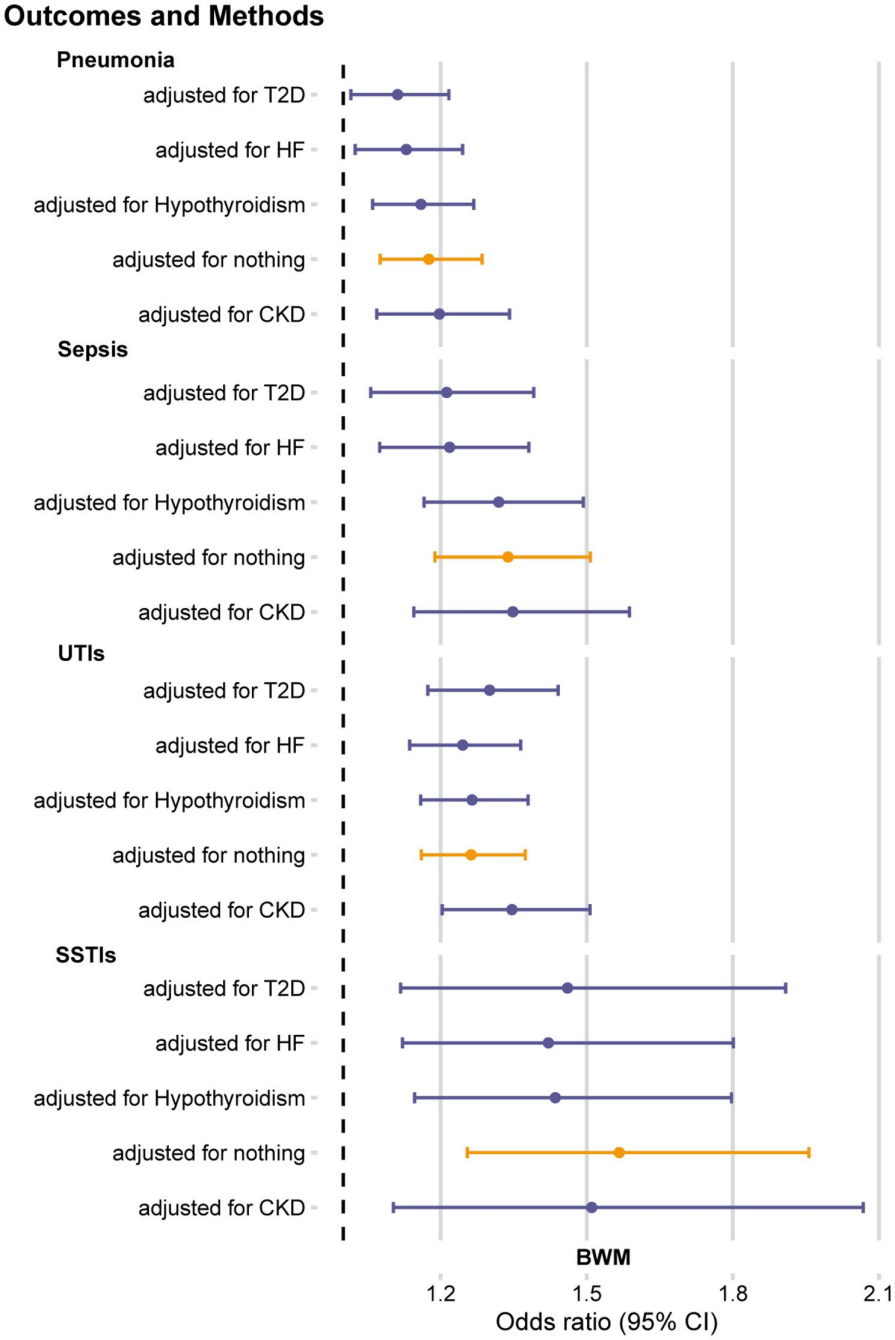
Multivariable MR analysis for associations of BWM with pneumonia, sepsis, UTIs and SSTIs BWM, Body Water Mass UTIs, Urinary tract infections SSTIs, skin and soft tissue infections CKD, Chronic Kidney Disease T2D, Type 2 Diabetes HF, Heart Failure

To further explore the impact of edema disorders on the association of elevated BWM with infections that show strong evidence, we performed a multivariable MR analysis. Factors including CKD, HF, T2D, and hypothyroidism were adjusted separately (Fig. 3). After adjusting for several edema disorders, genetically predicted BWM levels were similarly associated with sepsis, pneumonia, UTIs, and SSTIs (Supplementary Table 7). Body mass index and fat mass are well-known causal determinants of infectious disease risk. Subsequently, we further analyzed the MVMR of BWM on BMI and fat mass, and found that the relationship between BWM and infectious diseases may be related to BMI and fat mass (Supplementary Table 8). Supplementary Fig. 2 visualizes the results.

## Discussion

This MR study utilized large-scale GWAS summary data to investigate the causal relationship between BWM levels and the risk of several common infections. The results showed that genetically predicted BWM was associated with a higher risk of sepsis, pneumonia, UTIs, and SSTIs. In addition, genetically predicted BWM was also associated with a suggestive reduced risk of viral intestinal infections. MR analyses supported the risk of BWM for several edematous diseases. Multivariable MR analysis indicated that the risk of BWM and sepsis, pneumonia, UTIs and SSTIs remained after adjusting for edema diseases.

Elderly patients may initially require additional fluid intake to cope with dehydration, but comorbidities and age-related declines in organ function may increase vulnerability to overhydration, with attendant concerns about their increased risk of infection. Gonçalves et al. prospectively studied 48 critically ill patients with acute kidney injury (AKI) and provided convincing evidence that overhydration, as measured by bioimpedance, plays an important role in the prediction of survival in sepsis [7]. Recent evidence suggests that a significant increase in hydration derived from bioelectrical impedance at hospital admission is a significant predictor of failure to survive in patients with COVID-19 pneumonia [22, 23]. Clinical observations have shown that water deprivation does not reduce the oxygen content of subcutaneous tissue or wound infection [24]. However, too much water may lead to increased skin tension, resulting in blisters, skin breakdown and exudate extrusion, providing a favorable environment for bacterial infections. Although observational studies have shown that excessive body hydration is associated with an increased risk of infection and a poor prognosis, it is difficult to establish a causal relationship. MR analysis has grown in popularity as a powerful tool to explore the causal relationship between exposure and outcome using genetic variation. Our MR study further showed that high BWM levels are associated with the risk of sepsis, pneumonia, UTIs and SSTIs.

Increasing water intake can increase BWM levels in the short term. A previous prospective study showed that increasing water intake is an effective antimicrobial drug-sparing strategy to prevent recurrent cystitis in women [25]. It has also been shown that there is a lack of sufficiently valid evidence that increased hydration intervenes in the prevention of urinary tract infections [26]. This discrepancy may be related to confounders and reverse causality in epidemiologic studies. Our MR study overcame this concern and shower a correlation between BWM levels and increased risk of UTIs.

With the decline in physical function in aging, excess body fluids are common in the clinic, especially in the elderly. This condition is usually a result of water being retained in the body or the inability to get rid of excess water. Increased systemic water mass as a result of heavy drinking or over-hydration. Additionally, common chronic diseases such as heart failure, kidney failure, liver disease, hypothyroidism, and diabetes can cause the body to become more hydrated [27]. And the consequences of elevated water mass include heart failure, edema, hypertension, and even death. In our study, we found that BWM is causally associated with several diseases, including CKD, T2D, and heart failure, and these factors may be further associated with infection risk. Exploring the potential mediating role of these diseases could provide further clinical potential for BWM in predicting infection risk. The correlation between elevated BWM and infection risk has been limitedly explored in traditional observational studies. Our findings have important implications for body fluid management testing, as they bear the risk of leading to an increased risk of infection. Therefore, clinicians may need to be more attentive to the possibility of several infections in their patients while managing their fluids or monitoring their intake,

As stated above, the main strengths of our study are the reduction of confounding (BWM is influenced by chronic disease) and reverse causation bias (infections cause excessive BWM rather than BWM causing infections), and that it fills a gap in the question of increased BWM in infectious events. Measured BWM rather than verbal reports also have a higher degree of fidelity. However, there are several limitations: firstly, there is a lack of population diversity as the data were mainly conducted on European descent species. Additional research is needed to determine if it can be generalized to other populations. Second, because the infections analyzed were predominantly from hospitalized patients, some less serious infections, including community-onset, may have been excluded, and this component remains an important contributor. Again, there was overlap between exposure and the presence of certain infection traits and heterogeneity in some results, but the high F-statistics examined and the consistency of the results in the sensitivity analyses suggest that this phenomenon is unlikely to have a substantial impact on this study. Finally, we did not mediate the underlying mechanisms of this causal relationship. More research is needed in the future to elucidate the biology behind the relationship.

## Conclusions

In conclusion, these MR findings support that higher BWM is associated with an increased risk of sepsis, pneumonia, UTIs and SSTIs. Pathologic edema may be an important factor. Our study provides a theoretical basis for the use of BWM as a predictor of infection risk, and more biological mechanism studies are needed to explore novel interventions in the future.

### Electronic supplementary material

Below is the link to the electronic supplementary material.

Supplementary Material 1

Supplementary Material 2

Supplementary Material 3

## Data Availability

The GWAS dataset of whole body water mass analyzed in this study is available at https://gwas.mrcieu.ac.uk/datasets/ukb-a-267/, and GWAS datasets for infectious diseases are from the website: https://r9.finngen.fi/:, respectively. Summary-level data of COVID‐19 GWAS are available at the COVID‐19 Host Genetics Initiative website https://www.covid19hg.org/results/r7/. The dataset for edematous disorders in this study is available in the GWAS catalog at https://www.ebi.ac.uk/gwas/.

## Abbreviations

BWM: Body Water Mass
CIs: Confidence Intervals
GWAS: Genome-Wide Association Studies
IVW: Inverse-Variance Weighted; MR: Mendelian Randomization
OR: Odds Ratio
SNPs: Single Nucleotide Polymorphisms
BMI: Body Mass Index
UTIs: Urinary Tract Infections
SSTIs: Skin and Soft Tissue Infections
MVMR: Multivariable MR
