# Supplementary figures and images for: Mendelian randomization analysis reveals higher whole body water mass may increase risk of bacterial infections
Source: BMC Med Genomics. 2024 Jul 9;17:183. doi: 10.1186/s12920-024-01950-3 (PMC11232203; doi:10.1186/s12920-024-01950-3)

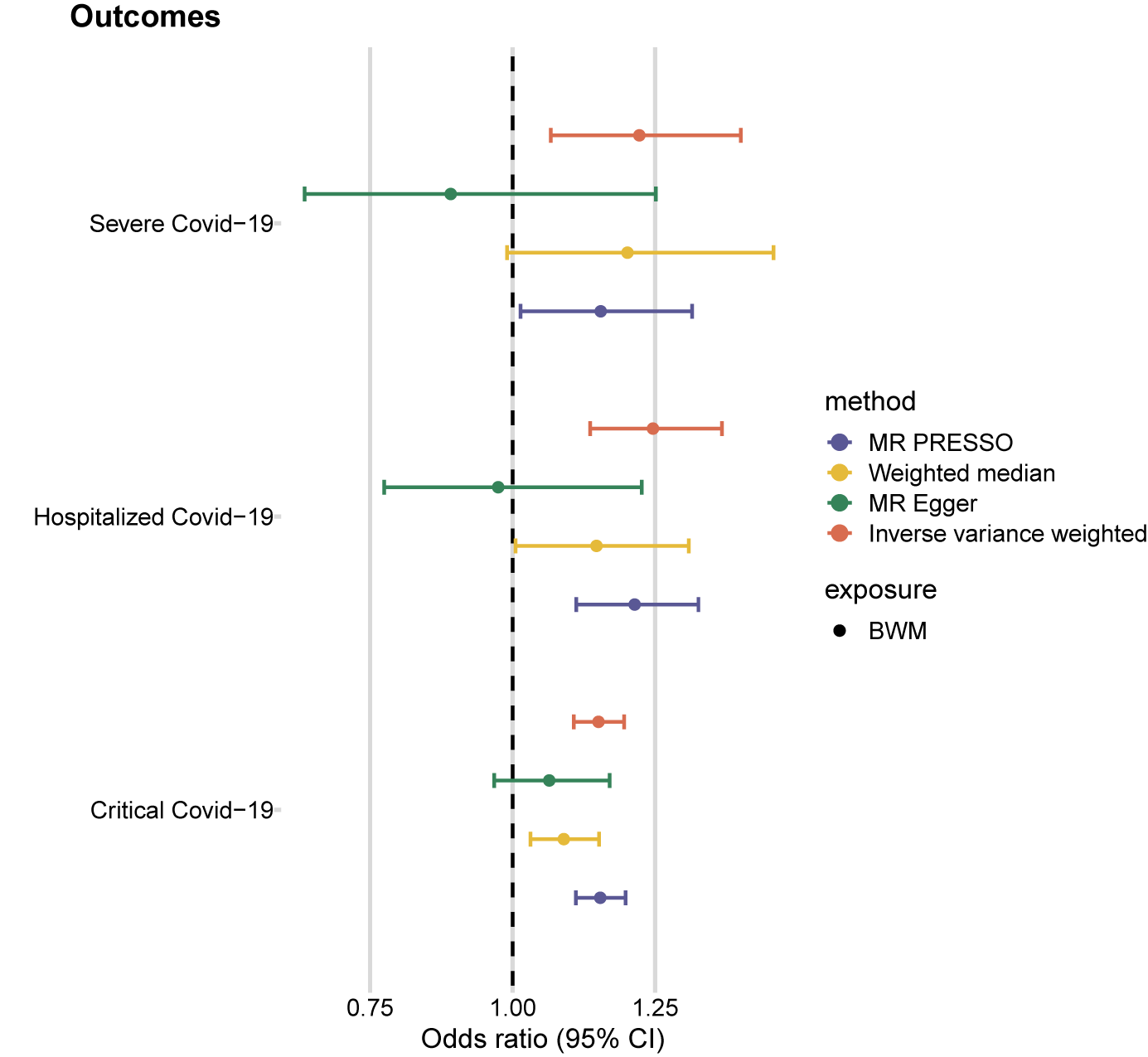

Supplement: Supplementary file 2 — Supplementary Material 2 [file 12920_2024_1950_MOESM2_ESM.tif]

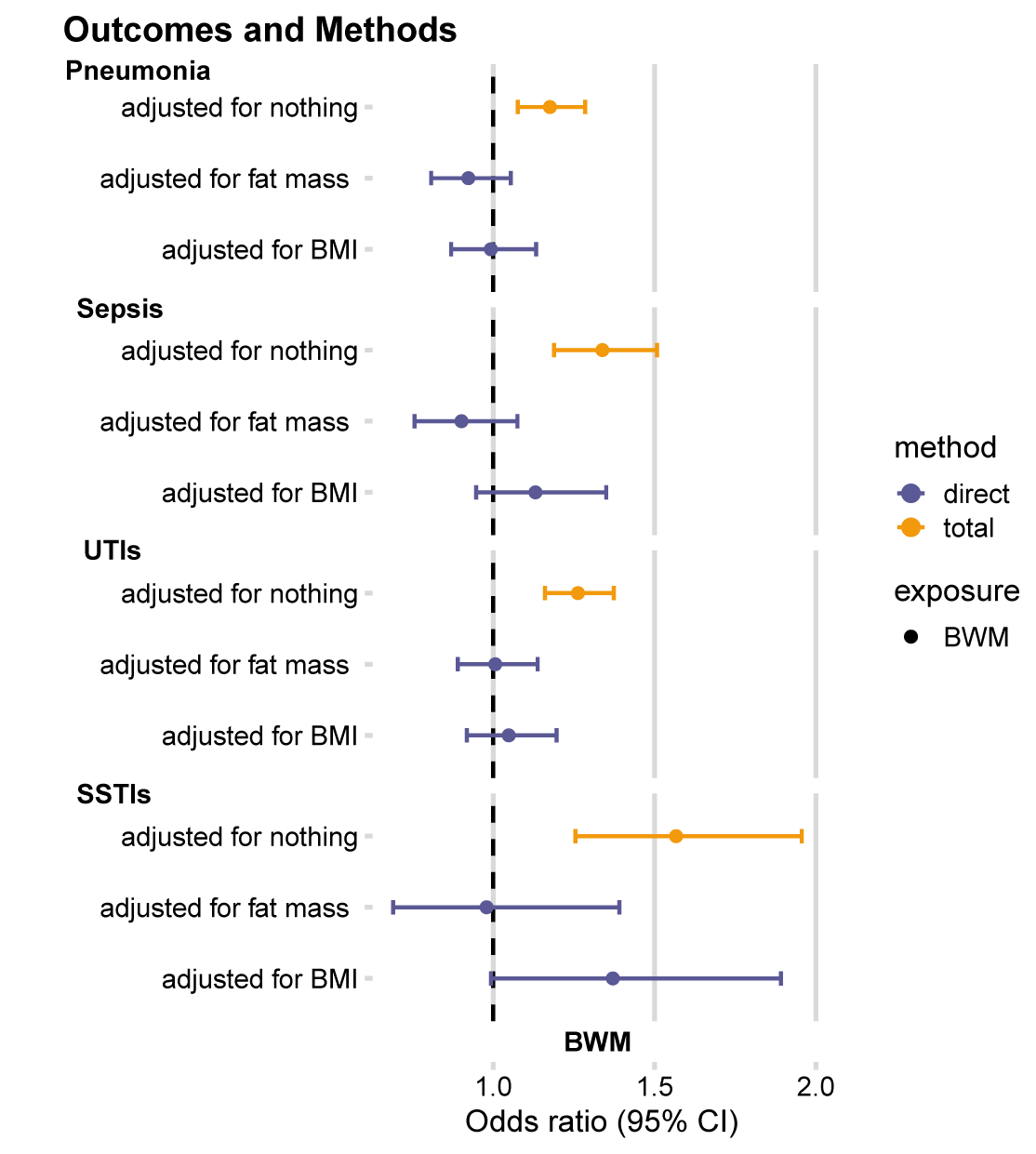

Supplement: Supplementary file 3 — Supplementary Material 3 [file 12920_2024_1950_MOESM3_ESM.tif]
